# Green-based anti-biofilm nanoformulations against bacterial contaminations in nosocomial environments

**DOI:** 10.1016/j.heliyon.2025.e42934

**Published:** 2025-02-23

**Authors:** Giovanni Lo Bello, Elena Dellacasa, Giacomo Damonte, Debora Caviglia, Anna Maria Schito, Roberto Raiteri, Orietta Monticelli, Marina Sartini, Maria Luisa Cristina, Laura Pastorino

**Affiliations:** a Department of Informatics, Bioengineering, Robotics, and System Engineering, University of Genova, Via Opera Pia 13, 16145, Genova, Italy; b Department of Chemistry and Industrial Chemistry, University of Genova, Via Dodecaneso 31, 16146, Genova, Italy; c Department of Health Science, University of Genova, Via Pastore 1, 16132, Genova, Italy; d Department of Integrated Surgical and Diagnostical Sciences, University of Genoa, Viale Benedetto XV 6, 16132, Genoa, Italy; e E.O. Ospedali Galliera, Hygiene Unit, Via Mura delle Cappuccine 14, 16128, Genova, Italy

**Keywords:** Nosocomial infections, *Staphylococcus aureus*, α-amylase, PCL nanoparticles, Antibiofilm nanoplatform

## Abstract

α-amylase enzyme molecules were conjugated to biocompatible and biodegradable *ad-hoc* synthetized polycaprolactone (PCL) nanoparticles, in order to fabricate an effective and innovative nanoplatform for the treatment of nosocomial *Staphylococcus aureus* contaminations. Indeed, PCL was chosen as the polymer matrix due to its features and the easy scalability of its synthesis, which enables to obtain a linear or star-shaped architecture with high functionality as in the polymer used.

The developed nanoformulation underwent extensive structural and functional characterization to evaluate nanoparticles size and morphology before and after enzyme immobilization. Its antibiofilm effectiveness was then validated against bacterial strains isolated from hospital surfaces, demonstrating its potential for practical anti-biofilm applications. The obtained results demonstrated that the system prepared from the enzyme-conjugated nanoparticles exhibited a significant enzymatic activity and an efficient ability to degrade the protective bacterial biofilm. The proposed nanoformulation can therefore be considered an effective and completely environmentally friendly material for surface disinfection in healthcare facilities, which can be safely used in different environment (e.g. sinks and pipes) or medical equipment surfaces (e.g. touch screens).

## Highlights

• Health care-associated infections cause significant mortality and healthcare costs.
• Pathogens can persist on surfaces forming protective biofilms enabling them to spread.
• α-amylase was used to disperse biofilms acting on their exopolysaccharidic component.
• Star-shaped polycaprolactone nanoparticles were *ad-hoc* synthetized.
• The presented green-based nanoplatform can be an innovative antibiofilm strategy.

## Graphical abstract

**Figure:**
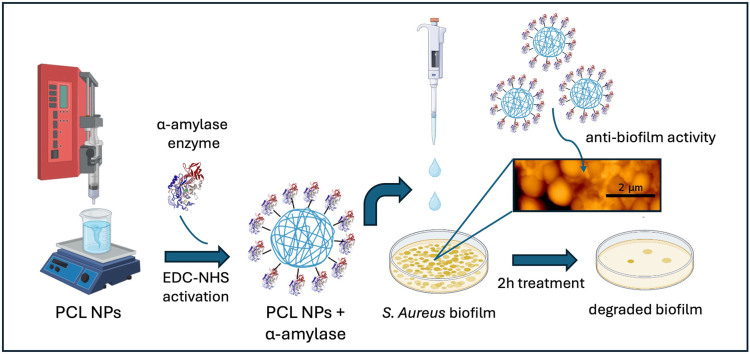

## 1 Introduction

Healthcare-associated infections (HAIs) are infections contracted by patients during their stay in a hospital or other healthcare settings that were not present or incubating at the time of admission.

Although some of these infections can be treated easily, others can have a more serious impact on patient's health, increasing mortality, hospitalization and hospital costs [1]. In the European Union and European Economic Area (EU/EEA), it is estimated that more than 3.5 million cases of HAIs (5.7%–7.1 % of all hospitalized patients) occur each year, leading to more than 90000 deaths and causing approximately 2.5 million disability-adjusted life years (DALYs) [2].

Among the microorganisms responsible for HAIs, *Staphylococcus aureus* (a human commensal bacterium involved in an array of pathologies, ranging from mild dermatological diseases to severe disorders, such as pneumonia, endocarditis, meningitis or sepsis) continues to be one of the main causes of hospital- and community-acquiredinfections worldwide [3,4].

Moreover, HAIs are increasingly caused by microorganisms that are resistant to first-line drugs and are often also multi-resistant; it is estimated that one in three infections in Europe is caused by antibiotic resistant microorganisms [5]. The hospital environment can play an essential role in the transmission of multidrug-resistant pathogens. The persistence of a nosocomial pathogen on surfaces in many hospital environments, such as patient rooms, can last for days, months, and even years, increasing the risk of transmission to the susceptible patient [6]. Strains of *Methicillin-Resistant S. aureus* (MRSA) have been detected on 1–27 % of surfaces sampled in patient rooms, but the presence of these microorganisms reaches 64 % in wards dedicated to the hospitalization of burn patients and in the presence of MRSA-positive patients. MRSA strains can remain viable on surfaces for more than 14 days and on cotton tissues for more than 6–9 weeks [7]. Of particular interest are the so-called high-touch surfaces, which serve as reservoirs for microorganisms, increasing the potential risk of cross-contamination [8]. Pathogens, including some strains of *S. aureus*, have also been shown to form biofilms on surfaces, allowing them to multiply, exchange antibiotic resistance genes with other bacteria, and survive for long periods of time within the protective matrix of the biofilm. Biofilms not only guarantee a stable adhesion to surfaces and provide structural support, but they also capture nutrients and act as shields against anti-bacterial treatments. Although the specific composition of this matrix, the extracellular polymeric substance (EPS), differs significantly among strains and under various growth conditions, its elements encompass extracellular DNA, polysaccharides and proteins [[9], [10], [11], [12]]. Nowadays, antibiofilm strategies are mainly based on two different approaches. The first consists of inhibiting or preventing biofilm formation, such as by using materials or coatings that exhibit chemical or structural anti-bacterial properties, and Quorum Sensing Inhibitors. For instance, surfaces coated with antibacterial materials, such as silver or copper alloys, can deter bacterial adhesion due to their inherent antimicrobial properties [13]. Structural modifications, such as superhydrophobic coatings, can further reduce bacterial attachment by minimizing surface contact [14]. Additionally, Quorum Sensing Inhibitors (QSIs), such as furanones or synthetic peptides, disrupt bacterial communication pathways, preventing the coordinated behaviors required for biofilm formation [15]. The second approach focuses on the dispersion or eradication of existing biofilms. Mechanical methods, such as high-pressure water jets, are commonly employed in food industries to physically remove biofilms from surfaces [16]. Ultrasound treatments, often combined with antimicrobial agents, enhance biofilm detachment by disrupting the extracellular polymeric substance (EPS) matrix through acoustic cavitation [17]. However, the complete eradication of biofilms is highly challenging due to their intrinsic properties. This scenario gives rise to the need to explore innovative strategies for the eradication of biofilm in healthcare settings. With regard to the development of innovative approaches in this field, the use of nanomaterials to combat bacterial biofilm associated infections has been widely studied [18,19]. Metallic nanoparticles such as silver, gold, copper, iron, and titanium have demonstrated remarkable antibiofilm and antibacterial properties [20,21]. These effects are attributed to a combination of physical mechanisms, such as membrane disruption, and chemical interactions, including the generation of reactive oxygen species (ROS), which collectively impair bacterial growth and biofilm integrity [22]. Moreover, polymeric nanoparticles have been proposed for the controlled release of antimicrobial agents or antibiotics or to disrupt bacterial membrane through electrostatic interactions [12]. However, despite the promising results obtained in this field, the biofilm still represents a protective shield to bacterial cells, greatly limiting the delivery of antibacterial molecules and nanoparticles [23].

To address this challenge, hydrolytic enzymes capable of degrading the main components of biofilms have been proposed as a strategy to enhance the penetration and efficacy of anti-biofilm and antibacterial agents [[24], [25], [26], [27], [28]]. Through the hydrolysis of the EPS components, these enzymes initiate the detachment of sessile bacterial cells, thereby increasing their susceptibility to antibacterial treatments. In this respect, DNase I has been widely studied to degrade extracellular DNA in biofilms and thus boost bactericidal efficiency when combined with other treatments [[29], [30], [31]]. However, the use of DNase I may be limited by its high cost compared with other enzymes [32].

Another component of EPS is represented by proteins, and various proteases with the ability to dissolve biofilms by degrading this component have been identified and studied so far [33,34]. However, the use of proteolytic enzymes may be limited in terms of combining the degradative approach with the use of antibacterial peptides [35]. Indeed, the main component of the EPS is represented by secreted extracellular polysaccharides, which play a crucial role in maintaining the integrity of biofilms. Various exopolysaccharides are found as components of bacterial EPS [36,37]. Among the different exopolysaccaride hydrolases tested so far, α-amylase has been shown to possess a strong inhibitory effect on *S. aureus* biofilm formation as well as a strong degradative activity towards pre-formed *S. aureus* biofilms [[38], [39], [40]]. Specifically, the α-amylase cleaves α-(1,4)-D-glycosidic linkage at random sites of amylose exopolysaccharide in biofilm matrices, resulting in oligosaccharides that lead to biofilm dispersing events. From an application point of view, α-amylase, being already used in food, fermentation, textile and paper industries [41], is interesting due to its thermostability, pH tolerance and low cost, making it a promising candidate for the combination of different treatment approaches [38,42].

Although the concept of using enzymes to promote the degradation of biofilms is not new, little has been reported on their immobilization for this application. Indeed, enzymes exhibit improved stability as a result of immobilization, which is an advantage for their storage and use [43,44]. Moreover, enzyme immobilization on nanoparticles provides additional advantages, including the high surface-area-to-volume ratio of the nanosystem, improved penetration into the biofilm matrix, and the ability to deliver multiple antimicrobial treatments simultaneously using a single nanocarrier [45]. To this purpose, α-amylase has been successfully immobilized mainly onto metallic nanoparticles [[46], [47], [48]], and onto the surface of medical devices or implants [49,50]. To the best of our knowledge, the application of nanoparticles functionalized with α-amylase for the dispersion of *S. aureus* is mainly referred to the treatment of infections *in vivo* [51].

The exploration of synthetic biopolymer nanoparticles as platforms for the immobilization of α-amylase for biofilm degradation has not been comprehensively documented compared to the more extensive research on the use of metal nanoparticles [52]. Indeed, synthetic biopolymers possess properties such as excellent mechanical stability, ease of modification, biodegradability and biocompatibility making them ideal for application as enzyme carriers [53,54].

This study presents the development and characterization of polycaprolactone (PCL) nanoparticles (NPs) functionalized with α-amylase, specifically designed for the degradation of *Staphylococcus aureus* biofilms in hospital environments. To achieve this, a custom-synthesized star-shaped polycaprolactone (PCL) with four arms, featuring carboxyl end groups (PCL-COOH), was employed to enable the covalent binding of α-amylase molecules. The NPs were characterized by field emission scanning electron microscopy, atomic force microscopy, infrared spectroscopy, dynamic light scattering and z-potential. The catalytic activity of the functionalized NPs was assessed by Ultraviolet–Visible Spectroscopy (UV–Vis), and the anti-biofilm activity was characterized by crystal violet assay, atomic force microscopy and fluorescent microscopy. A representative MRSA strain collected from nosocomial settings was used in this study. Overall, the results confirmed that the developed nanoplatform successfully degraded nosocomial *S. aureus* biofilms while significantly improving the thermal stability of the enzyme molecules. Due to its simplicity and to its composition, the nanoplatform could be perspectively used also for the delivery of antimicrobial molecules for the combined treatment of nosocomial surfaces.

## 2 Materials and methods

### 2.1 Materials

N-(3-Dimethylaminopropyl)-N-ethylcarbodiimide (EDC),N-Hydroxysuccinimide (NHS), α-amylase enzyme from Aspergillus oryzae, Polyvinyl Alcohol (PVA), Acetone, 4-Morpholinoethanesulfonic acid (MES), Phosphate Bufferd Saline (PBS), Tryptic Soy Broth (TSB), Crystal Violet dye (CV), soluble potato starch, Potassium Iodide (KI), Iodine (I_2_), Chloridic Acid (HCl), Poly(Diallyldimethylammonium chloride) (PDDA), Sodium Chloride (NaCl), ε-Caprolactone, Pentaerythritol, Tin(II) 2-ethylhexanoate (Sn(Oct)_2_), Methanol, Maleic anhydride, Toluene (anhydrous, purity 99,7 %), Dichloromethane (DCM, stabilized with 0,002 % 2-methyl-2-butene), DAPI, were all purchased from Merck. All the other reagents were of analytical grade and used without purification. Ε-Caprolactone and DCM were purified/dried prior to use by vacuum distillation over CaH_2_. The four-arm star-shaped PCL with carboxyl end groups (PCL-COOH) and arm of 2000 g/mol was synthesized according to the protocol developed by Damonte et al. [55,56]. Further details on polymer synthesis and corresponding ^1^H NMR and FT-IR assignments can be found in Damonte et al. [57].

The *S. aureus* strain under investigation was collected from environmental surfaces of inpatient rooms of a highly specialized hospital in northern Italy, using sterile swabs. The selected strain was previously subjected to molecular genotyping by spa typing technique and PFGE analysis and characterized as spa typing t002.

In addition, other three frequently encountered *S. aureus* strains (hereafter referred as A, B and C) were collected from different environmental surfaces of the hospital settings where the study was carried out and characterized as spa typing t002 (PFGE profile A), spa typing t032 (PFGE profile B) and spa typing t1312 (PFGE profile C). Theese strains were morphologically characterized by atomic force microscopy, to assess the antibiofilm capacity of the proposed nanoplatform also on different bacterial strains.

### 2.2 Methods

Polymeric NPs preparation and characterization has been reported in detail in section S1 of Supporting Information.

## 3 Results and discussion

**Fig. 1:**
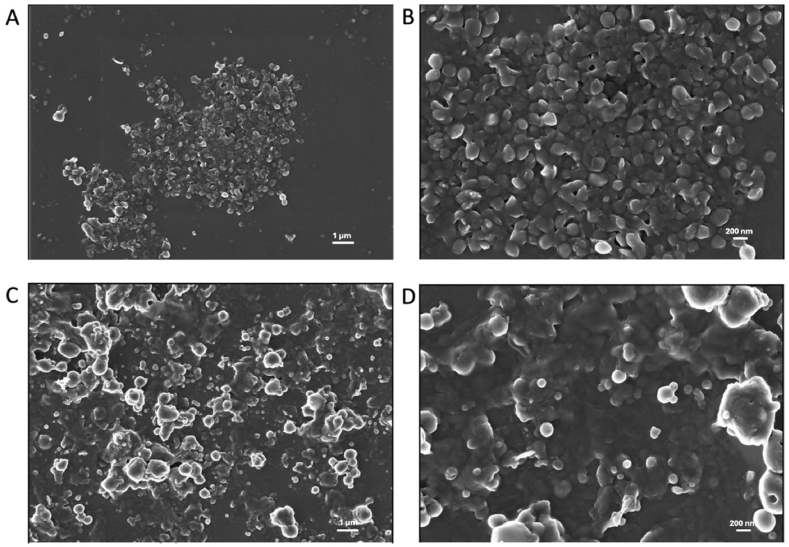
FE-SEM micrographs at different magnifications of plain (A, B) and functionalized (C, D) PCL NPs.

The morphology of NPs, based on the *ad-hoc* synthesized star-shaped PCL-COOH, was first investigated by FE-SEM at different magnifications. The micrographs in Fig. 1A and B shows that the plain NPs exhibite a spherical morphology and appear quite monodispersed, with an approximate diameter of 200 nm. Following enzyme immobilization, the NPs preserve their spherical shape but display a noticeable increase in polydispersity, which is consistent with the formation of micrometric aggregate (Fig. 1C and D). This increase in size and heterogeneity can be attributed to the conjugation of α-amylase molecules to the NPs’ surface, which may alter surface charge and induce particle–particle interactions [58,59]. However, despite this aggregation, the functionalized particles still fall within the nanoparticle size range, as their diameters remain below 1000 nm [60,61]. For our application, achieving a fully monodispersed nanoformulation was not strictly necessary, even though high monodispersity is generally desirable. Additionally, polymeric particles in biomedical contexts are used across a broad size range depending on the application and the biological barriers they are intended to overcome [[62], [63], [64]].

**Fig. 2:**
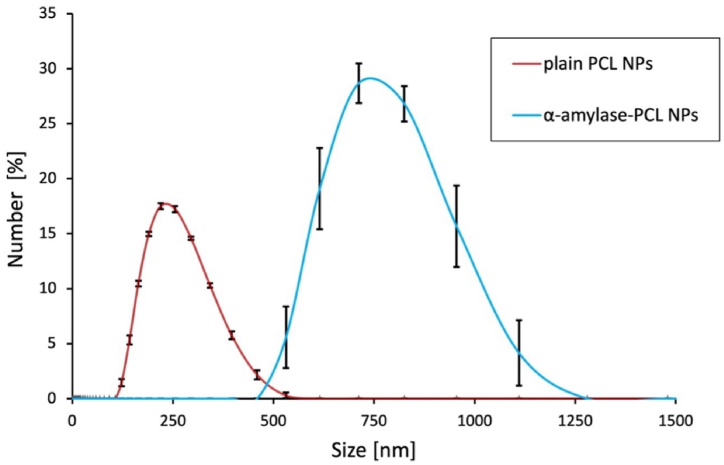
Size distribution of plain and functionalized PCL NPs.

DLS measurements were carried out to confirm and better quantify the results obtained by FE-SEM. The average diameter of plain NPs was (235.60 ± 76.71) nm, with a polydispersity index (PdI) of 0.16, confirming a narrow distribution. The average diameter of α-amylase-functionalized particles increased to a value of (807.20 ± 137.70) nm with a PdI of 0.25, indicating a wider size distribution, as shown in Fig. 2. The PdI is a measure of the sample heterogeneity based on its size and it is defined as the standard deviation (*σ*) of the particle diameter distribution divided by the mean particle diameter. From literature, it is well known that values of 0.2 and below are most commonly deemed acceptable for polymer-based nanoparticles [64]. Additionally, the observed agglomeration could result from the clustering effect of enzyme molecules during conjugation, a phenomenon that does not compromise their intended functionality but rather indicates successful surface functionalization. These results underscore the tradeoff between maintaining monodispersity and achieving effective functionalization of NPs with bioactive agents. The PdI generally impacts functionalities such as drug release *in vitro* or *in vivo* [63,65]. However, for this application, which focuses on maintaining enzyme stability and biofilm-degrading activity, the enzyme remains immobilized on the nanoparticle surface rather than being encapsulated or released, so PdI has a limited effect on functionality.

To better elucidate this phenomenom, ZP measurements were carried out on both plain and functionalized NPs. Moreover, since the conjugation of α-amylase to the particle surface should affect their surface charge, this characterization was also used to confirm the proper functionalization. The NPs showed differences in ZP values before and after the conjugation with the enzyme, with a mean ZP of (−41.60 ± 7.67) mV and (−23.50 ± 4.55) mV, respectively, confirming the modification of surface properties due to the enzymatic immobilization.

It is well known that ZP value higher than −30 mV and +30 mV generally confers sufficient repulsive force to attain better physical colloidal stability [[66], [67], [68]]. On the contrary, a small ZP value may cause the particle to tend to aggregate due to van der Waals attractive forces [69,70]. However, from the collected results, it can be assumed that both plain and functionalized NPs had sufficient ZP values to generate repulsive forces, even though their functionalization led to a slight increase in aggregation phenomena, which is also confirmed by the higher, but still acceptable PdI value [64,71].

AFM was used to characterized both plain and functionalized NPs at the nanometer scale. The height of the NPs extracted from topography AFM data was used to determine their diameter, under the assumption of uncompressible and spherical particles. The reason for this is that height measurements are not affected by tip-sample geometrical convolution as with lateral AFM measurements on the X-Y plane [72]. AFM imges confirm the same dimeter range observed by SEM measurements.

**Fig. 3:**
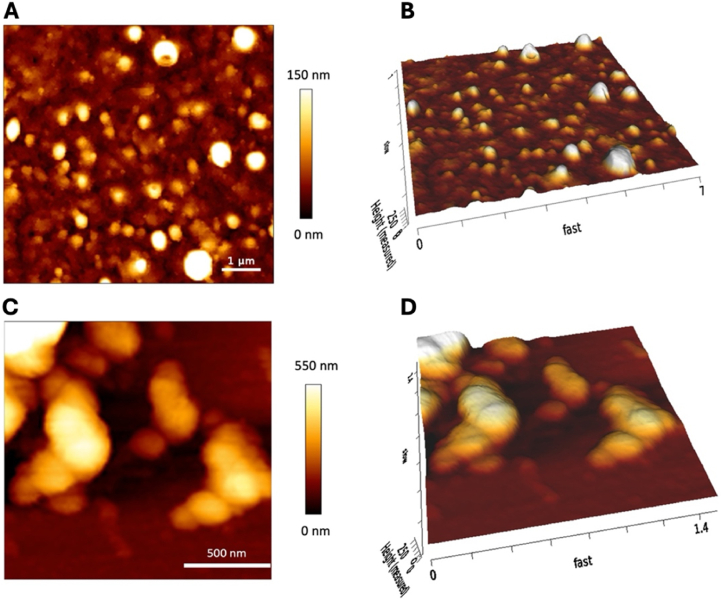
AFM morphological characterization of plain (A, B) and functionalized (C, D) PCL NPs.

The plain NPs showed a spherical and regular shape, as it is shown in Fig. 3A and B. Following enzymatic immobilization, NPs size increased up to 400 nm, as well as their aggregation (Fig. 3C and D), which produced large agglomerates with micrometric size (please see section Sof Supporting Informations for additional images). In conclusion, changings in diameter values can be attributed to the enzymatic functionalization. It is noticeable that diameters of functionalized NPs measured by DLS were higher than those measured by AFM and SEM. This can be due to the fact that DLS measures the hydrodynamic diameter of floating NPs dispersed in liquid, while SEM and AFM images refer to sedimented NPs.

**Fig. 4:**
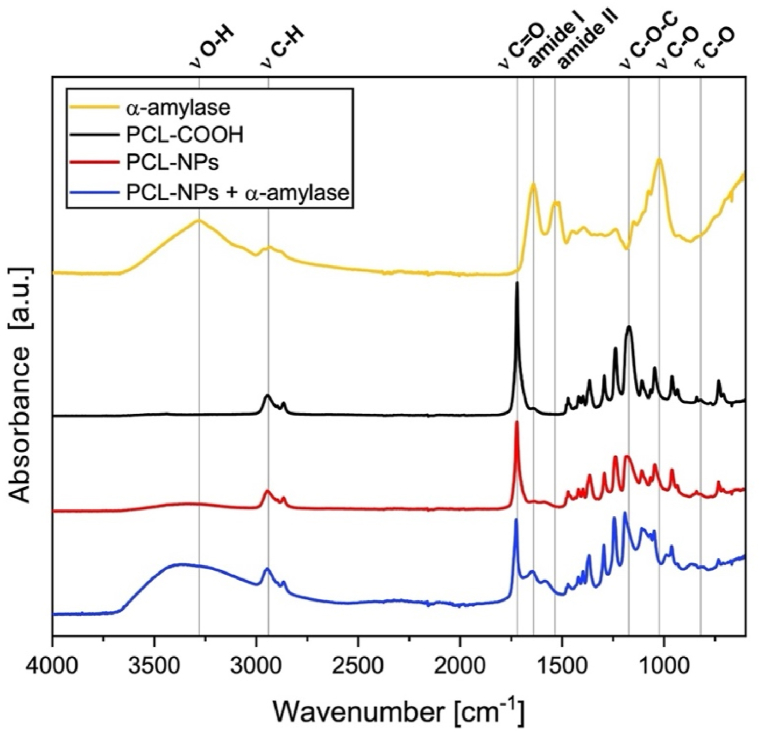
FT-IR spectra of: α-amylase, PCL-COOH polymer, plain PCL-NPs, and PCL NPs after conjugation with α-amylase.

Finally, to confirm the enzyme's coupling to nanoparticles, FT-IR spectra of the starting α-amylase powder and PCL-COOH were compared with those of the respective untreated (PCL NPs) and enzyme conjugated nanoparticles (NPs + α-amylase) (Fig. 4). Specifically, the FT-IR spectrum of α-amylase showed a series of peaks at 3300 (υ O-H), 2950 cm-1 (υ C-H), 1639 cm-1 (amide I band), 1534 cm-1 (amide II band), 1024 cm-1 (υ C-O and C-C), typical of polypeptide structures, in agreement with what has been reported by other authors for this enzyme [[73], [74], [75], [76]]. On the other hand, the analysis of neat PCL-COOH revealed a different spectrum with peaks at 2950 cm−1 and 2870 cm−1 (υ Csp3-H), 1720 cm−1 (υ C=O), at 1292 cm−1 (υ C–O and C–C), 1242 cm−1 and 1174 cm−1 (υ C–O–C) that were attributed to the PCL polymer backbone. Moreover, the presence of two additional signals in the spectrum, found at 1640 (υ C=C) and 814 cm-1 (τ C–O), ascribed to PCL-COOH maleic chain terminals, confirmed indirectly the presence of acidic moieties in the polymer [57,77,78]The analysis of pristine nanoparticles spectrum, PCL-NPs, displayed signals at 2950 cm−1 and 2870 cm−1 (υ Csp3-H), 1720 cm−1 (υ C=O), at 1292 cm−1 (υ C–O and C–C), 1242 cm−1 and 1181 cm−1 (υ C–O–C), which were consistent with the expected presence of PCL-COOH in the examined material. In contrast, the analysis NPs spectrum subjected to conjugation reaction, NPs + α-amylase, revealed the presence of signals at 3370 cm-1 (υ O-H), 2950 cm−1 and 2870 cm−1 (υ Csp3-H), 1725 cm−1 (υ C=O), 1645 cm-1 (amide I band), 1584 cm-1 (amide II band), 1295 cm−1 (υ C–O and C–C), 1242 cm−1 and 1192 cm−1 (υ C–O–C).

These peaks, previously found in the spectra of PCL-COOH and α-amylase, suggest the simultaneous presence of both substances in this sample. In particular, some of the characteristic enzyme's signals such as O-H stretching, amide I and II bands showed slight variations in terms of frequency and shape, which can be accounted to the interactions arising between α-amylase and the nanoparticles surface. In conclusion, these findings demonstrate that the conjugation procedure does not alter the molecular structure of the materials and confirm the actual presence of α-amylase on NPs only in bound form, as the sample was washed extensively to remove the unreacted enzyme.

Following the NPs structural characterization, the antibiofilm activity evaluation of free and conjugated enzymes was performed.

The catalytic activity tests were firstly performed with the aim at verifying α-amylase immobilization yield and its activity retention after immobilization. On this respect, α-amylase residual catalytic activity was found to be (14.02 ± 0.66) % with a (80.64 ± 0.36)% of immobilization yield. The low residual catalytic activity can be a consequence of working at room temperature for the catalytic activity evaluation, that it’s very far from the temperature that maximises α-amylase activity, but better replicate the environmental conditions [79]. In addition, as already mentioned above, catalytic activity loss can occur following the enzyme immobilization, depending on the chemical interactions between enzyme molecules and polymer functional groups [53,54]. On the contrary, it is also well known that the covalent immobilization of an enzyme can significantly increase its stability [62], as we also obtained from the subsequently presented results.

**Fig. 5:**
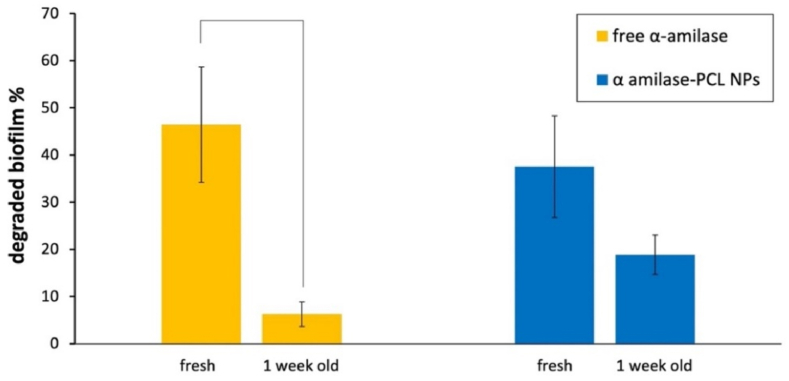
Antibiofilm activity of free α-amylase and α-amylase conjugated NPs comparison. Line indicates statistically significant difference (p < 0.01).

CV Assay was then performed to determine the MBEC of free α-amylase enzyme, indicated as the lowest concentration that produced visible disruption of biofilm. For this reason, 0.1 mg/ml and 0.4 mg/ml α-amylase concentrations were chosen for this study, supported by an accurate bibliographic research about of the most effective range of enzymatic concentrations in terms of biofilm reduction [[80], [81], [82]]. Specifically, 0.1 mg/ml and 0.4 mg/ml were able to degrade (45 ± 15)% and (41 ± 12)% of the selected *S. aureus* biofilm respectively. Considering the obtained results, no notable differences on the degradation percentages can be observed between the two concentrations, so 0.1 mg/ml was selected as MBEC and used for the further experiments with immobilized enzyme, as good compromise between shown antibiofilm activity and amount of functionalized NPs needed for each experimental session. Free α-amylase and α-amylase conjugated NPs antibiofilm activity was then compared and evaluated over time (Fig. 5).

As we can see from the graph in Fig. 5, fresh α-amylase conjugated NPs showed a biofilm degradation of (37.5 ± 10.8)%, comparable to that one of (46.4 ± 12.2)% obtained by the fresh free enzyme molecules, confirming a successful immobilization. Plain NPs didn't make any contribution to the biofilm eradication (data not shown).

The graph also compare the residual antibiofilm activity over time of fresh and one week old free enzyme and α-amylase conjugated NPs. As we can see from the percentage values, one week old α-amylase conjugated NPs show an activity loss corresponding to (18.7 ± 6.6) %, while α-amylase in its free form undergo to a statistically significant activity loss of (40.1 ± 9.6)% of its degradation activity (p < 0.01).

From these results, we can therefore assume a successful activity retention over time of α-amylase functionalized formulation, that after one week it's still able to degrade the (18.9 ± 4.2)% of biofilm, while the degradation capability of free α-amylase dropped to (6.3 ± 2.6)%.

**Fig. 6:**
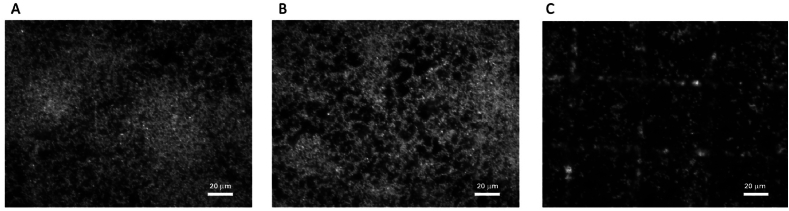
Fluorescence microscopy images of *S. aureus* biofilm as control (A), after plain PCL NPs interaction (B) and degraded by α-amylase conjugated NPs (C).

To exclude the contribution of plain PCL-COOH NPs on the antibiofilm activity, the selected *S. aureus* bioflm was grown and left in contact with plain NPs for 2 h at room temperature. The bacteria nuclei were then immunostained with DAPI in order to be characterized by fluorescence microscopy (Fig. 6).

In Fig. 6A a non-treated *S. aureus* biofilm is represented and used as a control. Fig. 6B clearly highlights that plain PCL NPs don't make a significant contribution to the biofilm degradation. On the contrary, Fig. 6C shows a notable biofilm eradication thanks to α-amylase enzyme conjugation, revealing the squared pattern of the glass coverslip with micrometric grid used as substrates.

**Fig. 7:**
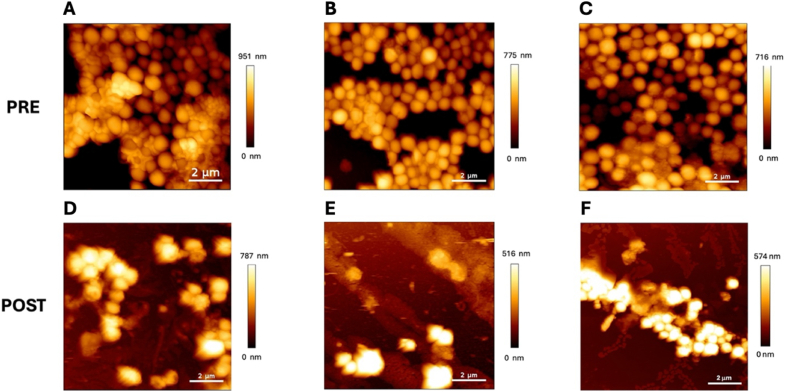
AFM topography images of three different 10 × 10 μm^2^ biofilm areas of selected *S. aureus* strain, pre (A, B, C) and post (D, E, F) treatment with α-amylase conjugated NPs.

Antibiofilm activity of α-amylase conjugated NPs was finally morphologically evaluated by AFM measurements (Fig. 7). First, 24h old bacterial biofilm was characterized before treatment, and three images of different rapresentatvie areas are shown in Fig. 7 A, B, C PRE (please see section Sof Supporting Information for additional images).

Bacteria showed the typical spherical shape, with a diameter between 300 nm and 1 μm. As can be seen from images, some of them appear as isolated clusters, while others were embedded within the EPS, displaying the characteristic non-homogeneity of biofilm structures.

After being characterized, *S. aureus* biofilm underwent a 2 h treatment with α-amylase conjugated NPs, and then washed with PBS. The same imaged areas were again characterized after treatment, thanks to the possibility to re-place the AFM probe with micrometrical accuracy (Fig. 7 D, E, F POST).

Biofilm morphology appeared clearly affected by the interaction with functionalized NPs, confirming a substantial eradication of most of the biofilm, leaving only few bacterial aggregates.

Finally, the biofilm eradication ability of the proposed nanoplatform was preliminarily evaluated on other three different environmental bacterial strains (namely A, B and C), to test the antibiofilm formulation efficacy dispite the aforementioned extrem variability of the EPS composition.

**Fig. 8:**
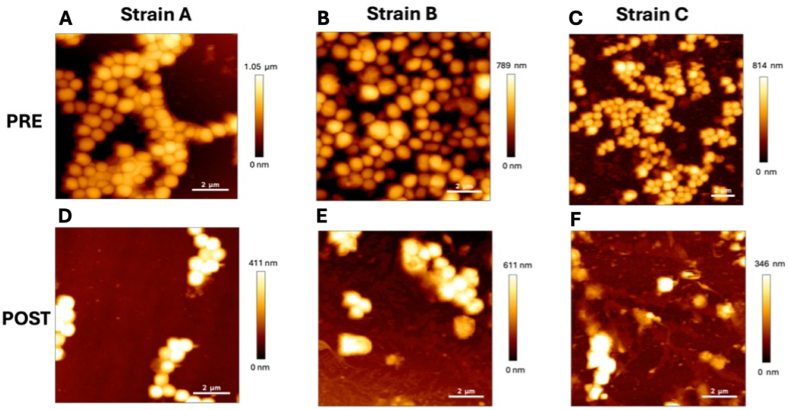
AFM topography images of three additional biofilms of *S. aureus* (namely A, B and C) pre (A, B, C) and post (D, E, F) treatment with α-amylase conjugated NPs.

To this purpose, AFM morphological characterization of pre (A, B, C) and post (D, E, F) treated biofilms was repeated and shown in Fig. 8.

Again, compared to the pre-treated biofilm (Fig. 8 A, B, C PRE) the post-treated biofilm structure (Fig. 8 D, E, F POST) appear noticeably altered, with a considerable decrease of bacterial density, confirming the effectiveness of this simple nanoformulation among different strains.

## 4 Conclusions

In this work, a novel anti-biofilm formulation based on a biocompatible and biodegradable polymer, namely PCL, was developed and its effectiveness was investigated. The results demonstrated the effective catalytic activity of the selected enzyme, namely α-amylase, towards the EPS matrix and the enhanced enzyme stability thanks to its immobilization on nanoparticles. Indeed, it was verified that the enzyme-conjugated NPs were able to degrade part of the polysaccharide components of the *S. aureus* biofilm, despite the high variability between samples and also the heterogeneity within each sample itself.

The developed formulation proved to be promising in terms of cleaning surfaces of nosocomial environmental settings and is worth to be further improved and tested not only on other *S. aureus* strains, but also on different microbial species, extending the research to those Gram negative microorganisms that are most implicated in HAIs. In this regard, the encapsulation of antimicrobial substances within the PCL-based NPs can make a useful contribution to the prevention of bacterial contamination by acting directly on the bacterial cells that are not protected by the EPS matrix.

## CRediT authorship contribution statement

**Giovanni Lo Bello:** Writing – original draft, Methodology, Investigation, Data curation. **Elena Dellacasa:** Writing – original draft, Validation, Methodology, Investigation, Data curation. **Giacomo Damonte:** Investigation, Data curation. **Debora Caviglia:** Methodology, Investigation. **Anna Maria Schito:** Methodology, Data curation. **Roberto Raiteri:** Supervision, Methodology. **Orietta Monticelli:** Writing – original draft, Supervision, Methodology. **Marina Sartini:** Writing – review & editing, Supervision. **Maria Luisa Cristina:** Writing – review & editing, Supervision, Funding acquisition, Conceptualization. **Laura Pastorino:** Writing – review & editing, Supervision, Funding acquisition, Conceptualization.

## Ethics declaration

Ethical approval was not required for this study because did not involve any direct experimentation/studies on living beings.

## Data availability statement

Data generated in this study are available in the manuscript along with supplementary file. For requesting additional data, please write to the corresponding author.

## Funding

This work was supported by 10.13039/100007388Fondazione Compagnia di San Paolo (ID ROL: 33687) and by supported by the 10.13039/501100000780European Union, PON Ricerca e Innovazione 2014–2020 D31B210 0815 0 0 01.

## Declaration of competing interest

The authors declare that they have no known competing financial interests or personal relationships that could have appeared to influence the work reported in this paper.
