# Supplementary material for: Green-based anti-biofilm nanoformulations against bacterial contaminations in nosocomial environments
Source: Heliyon. 2025 Feb 23;11(4):e42934. doi: 10.1016/j.heliyon.2025.e42934 (PMC11908580; doi:10.1016/j.heliyon.2025.e42934)
Supplement: Multimedia component 1 [file mmc1.docx]

**Supporting Information**

**GREEN-BASED ANTI-BIOFILM NANOFORMULATIONS AGAINST BACTERIAL CONTAMINATIONS IN NOSOCOMIAL ENVIRONMENTS**

**Giovanni Lo Bello, Elena Dellacasa, Giacomo Damonte, Debora Caviglia, Anna Maria Spagnolo, Anna Maria Schito, Roberto Raiteri, Orietta Monticelli, Marina Sartini, Maria Luisa Cristina, Pastorino Laura**

***S1. PCL-COOH NPs preparation and characterization***

**Polymeric NPs preparation**

Polymeric NPs were prepared using the solvent displacement technique as described by Fessi et al [1]. In brief, 2% w/v PVA (aqueous phase) was dissolved in pure water, stirred at 50°C for 2 hours, followed by 1 hour at room temperature, and then filtered through 0.2 µm syringe filters. Then, a solution of 5 mg/ml PCL-COOH in acetone (organic phase) was prepared. Subsequently, the organic phase was added dropwise to an equal volume of the aqueous phase using a syringe pump with a speed of 0.007 mL/min and a needle diameter of 22G. The mixture was stirred overnight at 650 rpm. Finally, the total volume was adjusted by adding pure water to the NPs dispersion to restore the initial PCL-COOH concentration. The NPs dispersion was first centrifuged at 1000 rpm at 4°C for 20 minutes to allow any micrometric aggregates to precipitate. Following that, two final washes at 11000 rpm for 20 minutes each were carried out, to remove any solvent residues. After washings, NPs were stored at 4°C for further characterization.

**Polymeric NPs functionalization**

Polymeric NPs were conjugated to α-amylase enzyme using EDC/NHS-assisted covalent binding method [2]. In summary, a covalent binding solution was prepared by dissolving 0.1 M EDC and 0.4 M NHS in 25 mM MES aqueous solution (pH 5). Subsequently, 250 μL of 5 mg/ml NPs dispersion were added to 1 mL of the covalent binding solution and gently shaken for 1 hour at room temperature. The activated NPs were then centrifuged twice at 15151 rcf for 20 minutes and a temperature of 4°C, to eliminate the unconjugated excess of EDC/NHS. The supernatant was replaced with 1 mL of α-amylase solution (1 mg/mL in 0.1M PBS), and NPs were gently shaken overnight at 4°C to immobilize the enzyme. Finally, the functionalized NPs were centrifuged at 15151 rcf for 20 minutes at 4°C to remove the excess of unconjugated enzyme, and the resulting waist washing was resuspended in PBS. Both functionalized NPs and washings were stored at 4°C.

The activity of both free and immobilized α-amylase was spectrophotometrically characterized using the starch–iodine assay, to assess the influence of the enzyme conjugation on its activity [3]. Briefly, 0.25 mg/mL starch solution was used as substrate, and an iodine reagent consisting of 2 mg/mL KI and 0.2 mg/mL I_2_ aqueous solutions was prepared for the starch staining. 400 μL of both free or immobilized α-amylase in 0.1 M PBS was added to 400 μL of the starch solution and allowed to react for 5 minutes at room temperature, to better replicate the environmental conditions. The reaction was terminated by adding 200 μL of 1M HCl and then 1 mL of iodine reagent was added to be spectrophotometrically characterized. The absorbance of the resulting blue coloured mixture was measured at 580 nm using a UV-vis spectrophotometer (Agilent Cary 60 Spectrophotometer).

The amount of conjugated enzyme to NPs was then measured according to the following equation (Eq. 1):

*Conjugated enzyme [µg] = free enzymatic solution [µg] - waist enzymatic solution [µg] (1)*

where the waist enzymatic solution represents the washing steps of the conjugation procedure.

To this purpose, the catalytic activity of the free enzyme solutions at different concentrations was determined, and the masses were calculated from the respective calibration curve for five standard solutions prepared by stepwise dilutions of the starting commercial solution (y = 31,692x - 0,1815, R^2^ = 0,9996).

The immobilization yield was then easily defined as the percentage of the ratio of the previously calculated conjugated enzyme to the initial free enzymatic solution (Eq. 2).

*Immobilization yield (%) = (Conjugated Enzyme [µg]) / (Stock enzymatic solution [µg] ) x 100 (2)*

Finally, the residual enzymatic activity of functionalized NPs was calculated as percentage (Eq. 3), in terms of actual conjugated enzyme, due to the possible partial loss of activity after immobilization [4,5] with respect to the expected conjugated enzyme (previously calculated with Eq.1), used as 100% value.

*Residual activity [%] = Actual conjugated enzyme [µg] / Conjugated enzyme [µg] x 100 (3)*

**NPs structural characterization**

Size and morphology of NPs were assessed using a Field Emission Scanning Electron Microscope (FE-SEM), specifically a Zeiss Supra 40 V P equipped with a backscattered electron detector. All samples underwent thin carbon sputter-coating using a Polaron E5100 sputter coater. FT-IR spectra were also recorded in ATR mode over the range of 400-4000 cm^−1^ using a Bruker “Vertex 70®”.

In addition, size distributions of plain and functionalized NPs and their surface potential were determined through Dynamic Light Scattering (DLS) and Zeta Potential, using a Zetasizer Nano ZS (Malvern Instruments, Worcestershire, U.K).

Finally, Atomic force microscopy (AFM) was used to image both plain and functionalized NPs. Specifically, NPs were allowed to precipitate on a previously functionalized mica sheet. Briefly, mica cleavage was followed by 100 μL of 1 mg/mL PDDA in 0.5M NaCl adsorption for 20 minutes. Then, several washes with pure water were carried out to impart a positive charge to the mica surface. Finally, 500 μL of 2.5 mg/mL NPs dispersion was deposited on the functionalized mica sheet for 4-5 hours to allow precipitation and surface adsorption before measurements. AFM images were obtained using a commercial microscope (NanoWizard 4 XP BioScience, Bruker, Billerica, MA, USA) equipped with a motorized stage for precise sample repositioning (HybridStageTM, Bruker), and coupled with an upright optical microscope (AxioZomm v.16, Zeiss, Jena, Germany). Topography images were obtained in liquid operating in the so called "Quantitative Imaging" (QI) mode and using a silicon nitride triangular cantilever with a sharpened tip (model MSCT-AUHW, Veeco Corp.). The cantilever deflection sensitivity was calculated before each laser re-alignment by pressing on a hard glass substrate. The cantilever spring constant K was determined by means of the Sader method [6], and was found to be 0.159 N/m. Images were collected at a resolution of 256x256 pixels, with a force setpoint in the range 0.5-1 nN, and a scanning speed between 40-80 um/s.

AFM images were processed using Version 6.0 of JPK NanoWizard® SPM software (Bruker, USA).

**NPs functional characterization**

To investigate the antibiofilm activity of α-amylase conjugated NPs, Crystal Violet (CV) assay and AFM were used.

Specifically, the ‘Minimal Biofilm Eradication Concentration’(MBEC) of free and conjugated enzyme was determined by CV assay [7]. First, free α-amylase enzyme was inoculated at two different concentrations, 0.1 mg/mL and 0.4 mg/mL, onto bacterial biofilms that had been growing for the previous 24 hours in 96 multiwell plate. Bacteria were previously thawed and plated on solid blood agar (Oxoid) plates for 24 hours to checked for viability and purity and subsequently placed in tryptic soy broth (TSB) (Oxoid) + 0.25% glucose and incubated at 37°C overnight. Samples were then diluted 1:100 in TSB + 0.25% glucose and inoculated in triplicate in sterile 96-well flat bottom plates and incubated at 37°C for 24 hours to allow biofilm formation. After this incubation the biofilms were gently washed 2 times with phosphate-buffer saline (PBS) to remove the non-adhered bacterial suspension. Then, three wells for each bacterial strains were left in contact with enzymatic solution for 2 hours at room temperature, while three wells were kept as control. At the end of the experiment, each well was washed twice with PBS, to remove the biofilm which was disrupted. Subsequently, CV dye was left to bind for 20 minutes to the remaining biofilm. After that, the unbound CV was washed off with three PBS washes and replaced with 90% ethanol to solubilize the coloured remaining biofilm. The multiwell plate was finally read by spectrophotometer at a wavelength of 570 nm, to quantify the percentage of still attached biofilm. The percentage of removed biofilm was then calculated, as the difference between the still attached biofilm percentage and the control biofilm used as 100%.

The same CV assay was repeated to test the antibiofilm activity of both plain and α-amylase functionalized NPs dispersed in PBS, using the predetermined MBEC as reference, and PBS solution as control. The concentration of functionalized nanoparticles was calculated to have the same amount of α-amylase both in its immobilized and free form.

Antibiofilm activity of plain NPs were also characterized by fluorescence microscopy, to certainty attribute the biofilm degradation to the enzymatic conjugation. Biofilms were left in contact with both plain and functionalized NPs for two hours. After washings, samples were fixed in 4% paraformaldehyde in phosphate buffer solution (0.01 M PBS), pH 7.4 for 30 min at room temperature. Permeabilization was achieved with PBS containing 0.1% Triton- X100 for 15 min at room temperature and non-specific binding of antibodies was blocked with an incubation of 45 min in a blocking buffer solution consisted of PBS, 0.3% BSA (bovine serum albumin) and 0.5% FBS. Samples were incubated with DAPI 1:5000 diluted in PBS Blocking buffer for 10 min at room temperature, rinsed three times with PBS and then characterized.

Functionalized NPs antibiofilm activity was then investigated at the nanoscale level by AFM measurements, before and after the interaction with conjugated α-amylase NPs, leading to a qualitative evaluation of the biofilm eradication. To this purpose, 24-hour old *S. aureus* biofilms were grown in 40 mm petri dishes, following the same protocol described. AFM images were recorded in air, using the same scanning parameters used for the NPs samples and described in the previous paragraph. In order to scan the same region of the biofilm, before and after a 2-hour long treatment, a glass coverslip with a 50 μm pitch distance grid (Ibidi GmbH, Germany) was used as substrate. The ruled grid allowed to visually reposition the AFM probe on the same scanning area before and after treatment (please see further details in section S2 of Supporting Information). In addition, using the "direct overlay" feature of the AFM software, it was possible to select the scan area directly onto the optical image with an accuracy of a few micrometres.

Finally, free and conjugated α-amylase antibiofilm activity was also compared and evaluated over time by CV assay. Free enzymatic solution and enzyme conjugated NPs were both prepared and used fresh and after one week at 4° C conservation.

All experiments were performed in triplicate and results are shown as mean ± standard deviation.

For statistical analyses, P values were determined by using a Student t-test performed with Microsoft Excel software.

***S2. AFM characterization***

AFM images of both NPs and biofilm were recorded using specifically modified Petri dishes.

In particular, 40 mm Petri dishes were pierced in the centre, in order to place 1.5 mm thick gridded round coverslips, with a diameter of 28 mm (Figure S1). Each grid of size 500 µm x 500 µm (Namely A, B, C and D) were further divided in smaller 50 µm x 50 µm grids. Both Petri dishes and coverslips were previously washed in an ultrasound bath with 5% soap water solution and then dried in the oven at 60 °C. Finally, the modified Petri dishes were washed with 70% ethanol solution and sterilized under UV rays.


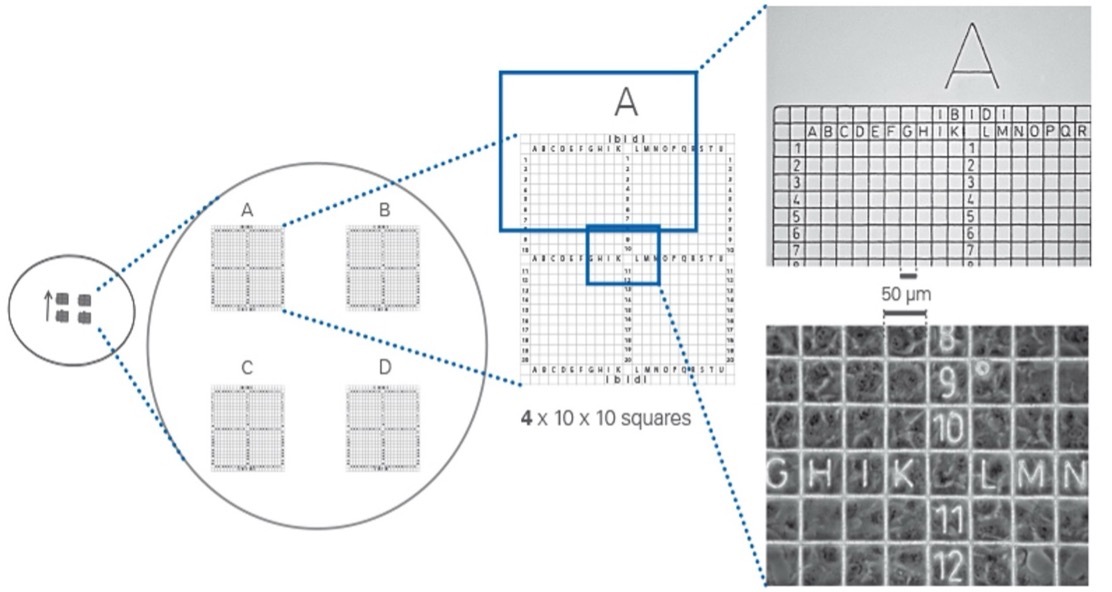


**Figure S1:** Ibidi gridded glass coverslip used for AFM characterization

As illustrative examples, AFM images of NPs and *S. aureus* biofilm are presented below.

Figure S2 depicts a cross-sectional view of a plain and functionalized nanoparticles, where the functionalized nanoparticles exhibit a slight increase in average size compared to the plain ones.


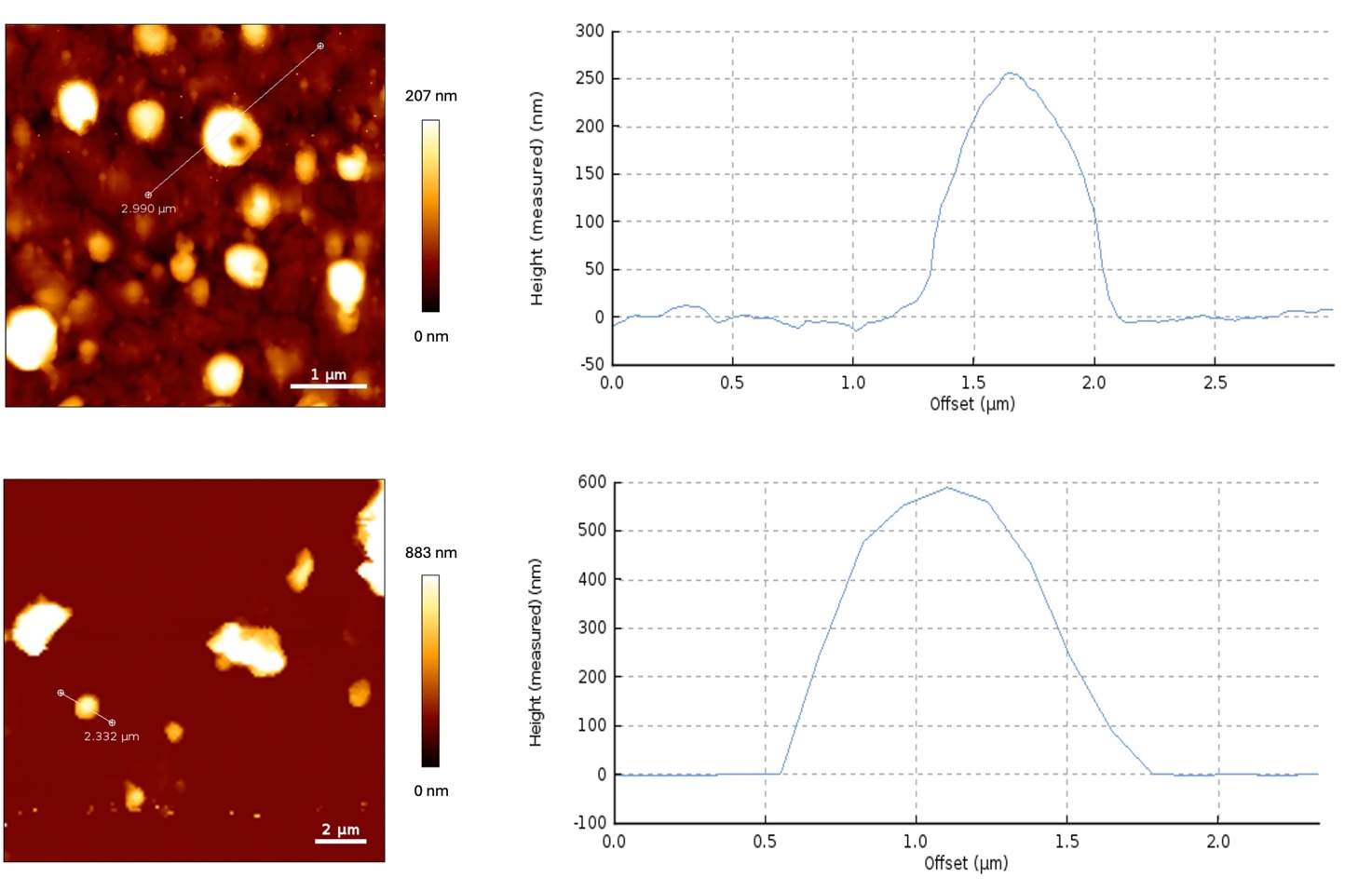


**Figure S2**: AFM morphological characterization of plain (top) and functionalized (bottom) nanoparticle and their cross section

Finally, Figure S3 presents a cross-sectional view of two adjacent bacteria, highlighting a height dimension of approximately 400 nm, encompassing both smaller and larger sizes.


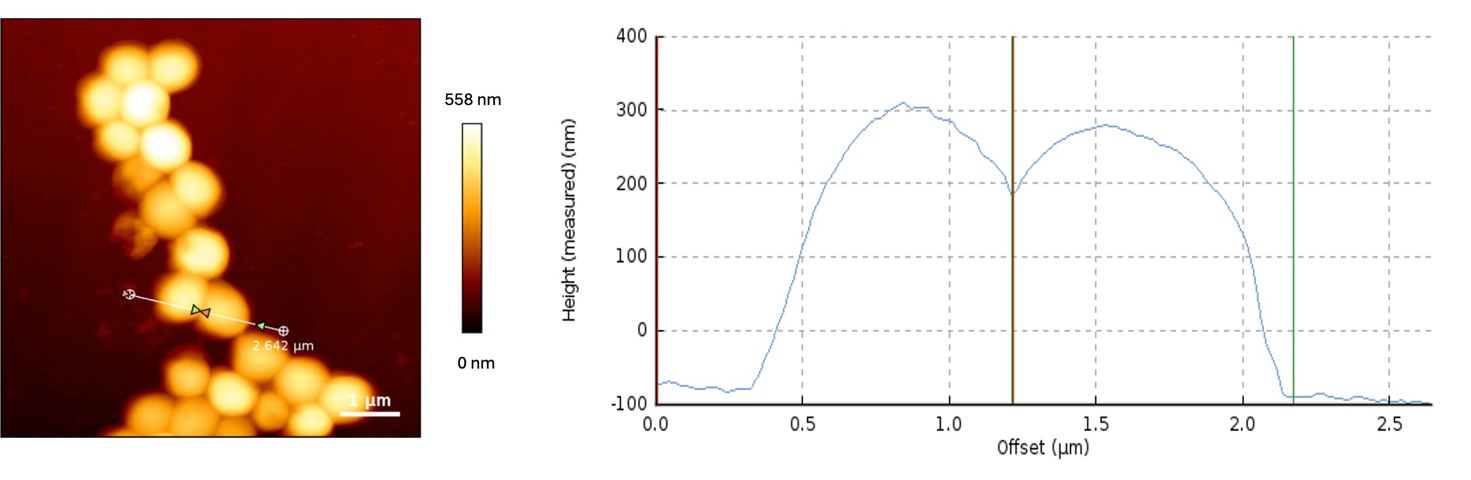


**Figure S3**: AFM morphological characterization of *S. aureus* biofilm (left)and the cross section of two adjacent bacterial cells (right)

**References**

[1] H. Fessi, F. Puisieux, J.P. Devissaguet, N. Ammoury, S. Benita, , Interantional Journal of Pharmaceutics 55 (1989), <https://doi.org/10.1016/0378-5173(89)90281-0>.

[2] E. Turan, Hittite Journal of Science & Engineering (2018). https://doi.org/10.17350/HJSE19030000081.

[3] Z. Xiao, R. Storms, A. Tsang, Anal Biochem 351 (2006) 146–148. <https://doi.org/10.1016/j.ab.2006.01.036>.

[4] G. Damonte, A. Vallin, D. Battegazzore, A. Fina, O. Monticelli, React Funct Polym 167 (2021). https://doi.org/10.1016/j.reactfunctpolym.2021.105019.

[5] G. Damonte, B. Barsanti, A. Pellis, G.M. Guebitz, O. Monticelli, Eur Polym J 176 (2022). https://doi.org/10.1016/j.eurpolymj.2022.111402.

[6] J.E. Sader, J.W.M. Chon, P. Mulvaney, Review of Scientific Instruments 70 (1999) 3967–3969. <https://doi.org/10.1063/1.1150021>.

[7] S. E. Cramton, C. Gerke, F. Götz, Methods Enzymol. 336 (2001) 239-55. doi:

10.1016/s0076-6879(01)36593-x.
